# An immunomodulatory photosensitizer-mediated photodynamic therapy synergizes with PD-L1 blockade against metastatic triple-negative breast cancer

**DOI:** 10.3389/fphar.2025.1651165

**Published:** 2025-07-17

**Authors:** Yuetong Liu, Liming Wang, Feiyan Kong, Tianjun Liu, Hong Liu

**Affiliations:** ^1^The Second Surgical Department of Breast Cancer, Tianjin Medical University Cancer Institute and Hospital, National Clinical Research Center for Cancer, Tianjin, China; ^2^ Tianjin’s Clinical Research Center for Cancer, Tianjin, China; ^3^Key Laboratory of Breast Cancer Prevention and Therapy, Ministry of Education, Tianjin Medical University, Tianjin, China; ^4^State Key Laboratory of Component-Based Chinese Medicine, Haihe Laboratory of Modern Chinese Medicine, Instrumental analysis and Research Center, Tianjin University of Traditional Chinese Medicine, Tianjin, China; ^5^School of Basic Medical Sciences, Hebei University, Baoding, China; ^6^State Key Laboratory of Advanced Medical Materials and Devices, Tianjin Key Laboratory of Biomedical Materials, Institute of Biomedical Engineering, Chinese Academy of Medical Science and Peking Union Medical College, Tianjin, China

**Keywords:** photodynamic therapy, immunogenic cell death, metastatic triple-negative breast cancer, programmed death-ligand 1 blockade, abscopal effect, systemic antitumor immunity

## Abstract

The clinical potency of anti-programmed death-ligand 1 (PD-L1) therapy in metastatic triple-negative breast cancer (TNBC) is modest primarily because of the intrinsic low immunogenicity and an immunosuppressive tumor microenvironment (TME). Photodynamic therapy (PDT), an inducer of immunogenic cell death (ICD), has the potential to enhance antitumor immune response and improve PD-L1 blockade efficacy. DTP, a novel photosensitizer developed previously, has demonstrated potent ROS-dependent photocytotoxicity, yet its immunomodulatory effects remain unexplored. This study investigated the induction of ICD and dendritic cell (DC) maturation following DTP-PDT *in vivo* and *in vitro*. A bilateral TNBC model was developed to assess the efficacy of DTP-PDT combined with α-PD-L1 therapy on untreated distant tumors and to explore its potential immunological mechanisms. The results showed that DTP-PDT effectively induced ICD, demonstrated by calreticulin membrane exposure, high mobility group box 1 protein release, and increased secretion of interferon-γ and tumor necrosis factor-α, resulting in DC maturation. The combination of DTP-PDT and α-PD-L1 significantly inhibited distant tumor growth. This effect was associated with increased CD8^+^ and CD4^+^ T cells infiltration, and reduced numbers of regulatory T cells, in the distant tumor and spleen. In conclusion, DTP-PDT enhanced TNBC sensitivity to α-PD-L1 by inducing ICD, and its combination withα-PD-L1 could remodel the immunosuppressive TME and enhance systemic immunity, resulting in a therapeutic effect against distant metastasis. This study provides experimental validation for a combined strategy of DTP-PDT and α-PD-L1, proposing a potential therapeutic approach for metastatic TNBC.

## 1 Introduction

Triple-negative breast cancer (TNBC) represents 15%–20% of breast cancer cases (Garrido-Castro et al., 2019) and demonstrates aggressive biological behavior, lack of therapeutic targets, and a tendency for early metastasis (Criscitiello et al., 2012; Leon-Ferre and Goetz, 2023). Current treatments for TNBC primarily include surgery, chemotherapy, and radiotherapy (Subhan, 2024). While these approaches are effective in early-stage TNBC, they offer limited success against metastatic TNBC (mTNBC) (Li Y. et al., 2022).

Cancer immunotherapy, which involves engineering immune cells to specifically target and eliminate tumors, has attracted widespread attention over the past decade, especially for metastatic cancers (Kennedy and Salama, 2020; Pham et al., 2021). In mTNBC, immune checkpoint inhibitors, particularly the programmed death-1/programmed death-ligand 1 (PD-1/PD-L1) blockade, have emerged as a key immunotherapeutic approach (Zhu et al., 2023). However, PD-L1 blockade monotherapy yields suboptimal outcomes even in PD-L1-positive patients, with objective response rates (ORR) of 40% and median response durations less than 12 months (Schmid et al., 2018; Cortes et al., 2020).

The limited efficacy of PD-L1 blockade is largely due to two main factors: the low immunogenicity characterized by insufficient tumor antigen release and presentation; and the “cold” immunosuppressive tumor microenvironment (TME) (Liu et al., 2023). To address these challenges, current research is focused on enhancing tumor immunogenicity and transforming the “cold” TME into a more immunogenic environment to improve responses to immunotherapy (Khosravi et al., 2024). Emerging evidence underscores that combination therapies targeting multiple stages of the cancer immune cycle may offer enhanced therapeutic efficacy of PD-L1 blockade (Meric-Bernstam et al., 2021). These strategies include chemotherapy, anti-angiogenic agents, immune modulators, and localized treatments such as radiotherapy and photodynamic therapy (PDT) (Zhu et al., 2021).

PDT is a localized treatment that functions by activating photosensitizer with specific wavelength light, triggering the production of reactive oxygen species (ROS) which induce tumor cell death (Agostinis et al., 2011). Compared to conventional therapies, PDT provides distinct benefits, including minimal invasiveness, low systemic toxicity, and high selectivity (Brown et al., 2004; Pham et al., 2021). Recent advancements have highlighted that certain photosensitizers not only eradicate tumors locally but can also induce the releases of tumor-associated antigens and damage-associated molecular patterns (DAMPs), thereby stimulating systemic antitumor immunity (Li Z. et al., 2022). Preclinical studies in melanoma (Li et al., 2025), lung (Zhao et al., 2023), and pancreatic cancer (McMorrow et al., 2025) models have demonstrated that combining this immunogenic effect with PD-L1 blockade can overcome therapeutic resistance and enhance systemic immune responses. Additionally, PDT disrupts endothelial structures and increases vascular permeability, improving the intratumoral delivery of α-PD-L1 antibodies (Bhandari et al., 2024). Some studies have also shown that PDT can upregulate PD-L1 and PD-1 expression on lymphocytes (Lobo et al., 2023)or downregulate PD-L1 on tumor cells (He et al., 2024). In clinical settings, the combination of PDT and PD-L1 blockade has been found to remodel antitumor immunity in gastric cancer patients by increasing cytotoxic T lymphocytes (CTLs) infiltration and suppressing regulatory T cell (Treg) activity, ultimately leading to improved overall survival outcomes (Yu et al., 2023). These findings highlight the potential of PDT-based combination strategies to transform the immunosuppressive TME and enhancing immunotherapy efficacy. However, conventional porphyrin-based photosensitizers, despite serving as the foundation for PDT due to their efficient light absorption capabilities (Arnaut, 2011), face significant challenges including limited water solubility, inadequate tumor specificity, and pronounced dark toxicity (Tian et al., 2020; Akbar et al., 2023; Yin et al., 2024).

To overcome these challenges, Professor Tianjun Liu’s team developed a novel porphyrin-based photosensitizer, Meso-5-[ρ-diethylene triamine pentaacetic acid-aminophenyl]−10,15,20-triphenyl-porphyrin (DTP). DTP demonstrates excellent water solubility and significant phototoxicity when irradiated with a 650 nm laser in various cancer cell lines, while exhibiting low dark toxicity (Chen et al., 2015; Chen et al., 2016; Chen et al., 2017; Chen et al., 2024). These properties make it a promising candidate for further investigation.

**FIGURE 1:**
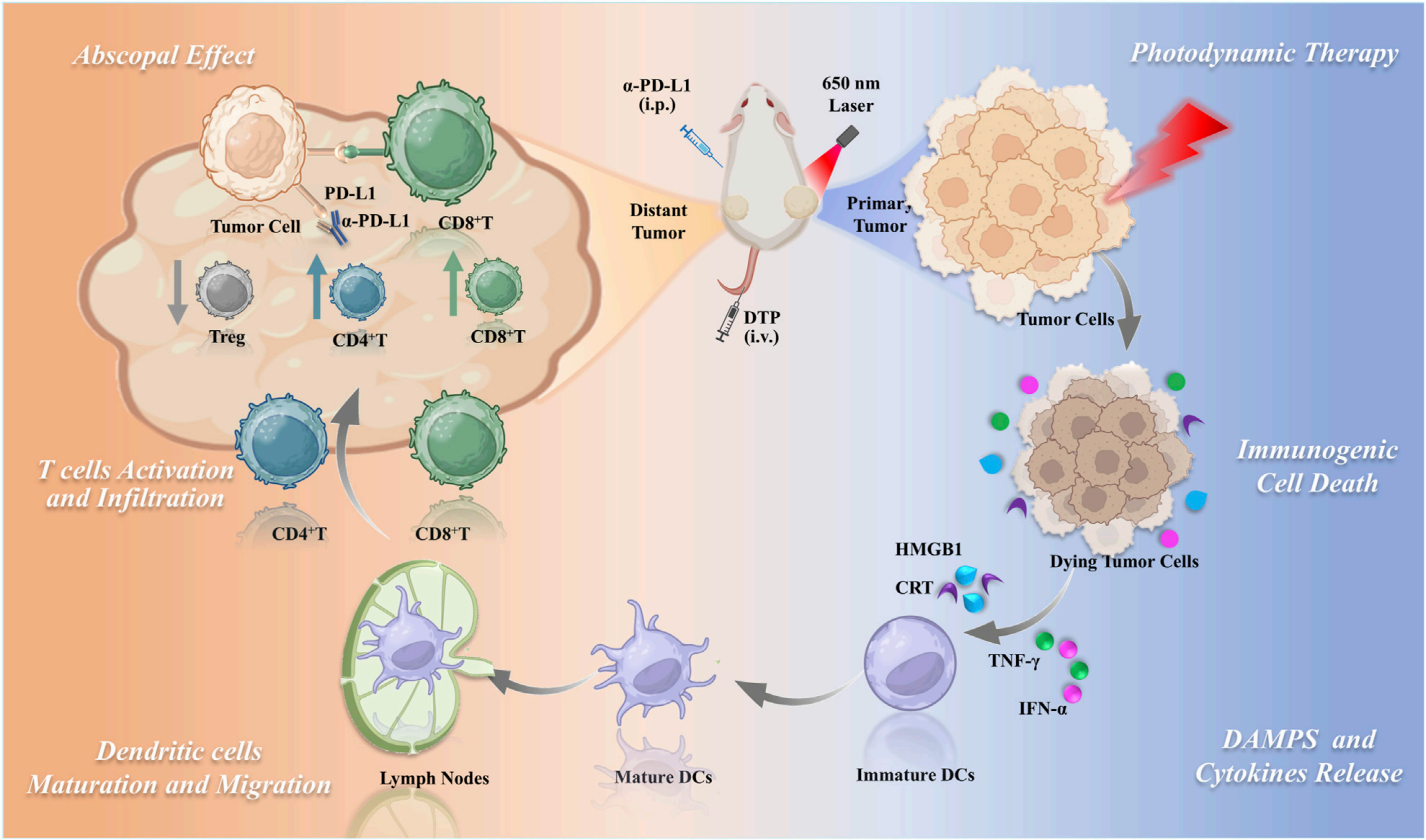
Schematic of DTP-PDT combined with PD-L1 blockade for enhanced systemic antitumor immunity in triple-negative breast cancer.

Building on the promising photodynamic properties of DTP and considering the potential of PDT to improve immunotherapeutic responses, this investigation was designed to assess the capacity of DTP-PDT to elicit antitumor immunity. Additionally, this study explores the synergistic effect and underlying mechanisms of combining DTP-PDT with PD-L1 inhibitors for the treatment of mTNBC (Figure 1).

## 2 Materials and methods

### 2.1 Photosensitizer and light source

The photosensitizer DTP was synthesized and provided by Prof. Tianjun Liu (Institute of Biomedical Engineering, Chinese Academy of Medical Sciences and Peking Union Medical College). The purity of DTP was over 98% by High Performance Liquid Chromatography. UV-Vis absorption profiling of DTP dissolved in dimethyl sulfoxide (DMSO) was performed on a UH5700 spectrophotometer (Hitachi, Japan). A 650-nm semiconductor laser (WSLS-650-500m-M-2; Wave Spectrum Laser Group Limited, China) via a columnar fiber was used in the *in vivo* and *in vitro* study, ensuring precise and controlled irradiation conditions. The energy density of the illuminated spot was quantified using a light power meter (Coherent Corp., PA, United States).

### 2.2 Cell culture

The murine 4T1 breast tumor cell line was sourced from the National Collection of Authenticated Cell Cultures (Chinese Academy of Sciences, Shanghai). Cells were cultured in RPMI 1640 medium containing 10% fetal bovine serum (FBS) and 1% penicillin-streptomycin (PS), under standard humidified incubation (37°C, 5% CO_2_).

### 2.3 *In vitro* ICD biomarker analysis

4T1 cells plated in confocal dishes underwent 24-h incubation with DTP (200 nM). Subsequently, cultures were irradiated (650 nm laser, 20 mW/cm^2^, 5 min) or maintained as non-irradiated controls. Post-treatment, cells were fixed in 4% paraformaldehyde (PFA) (15 min, RT), permeabilized in 0.1% Triton X-100 (high mobility group box 1 (HMGB1) detection only), and blocked with 1% bovine serum albumin. Primary antibodies include anti-calreticulin (CRT) (1:200, ABclonal, A1066) or anti-HMGB1 (1:200, Wanleibio, WL03023) were applied overnight (4°C), followed by Alexa Fluor 488-conjugated secondary antibody incubation (1.5 h, RT). Cells were counterstained with DAPI (5 min, RT) and specimens imaged by confocal laser scanning microscopy (CLSM).

### 2.4 Isolation and culture of bone marrow-derived dendritic cells (BMDCs)

Bone marrow was isolated from femurs and tibias of 5–7-week-old C57BL/6 mice. Following erythrocyte lysis (Beyotime, C3702), cells were suspended in RPMI 1640 medium supplemented with 10% heat-inactivated FBS, 1% PS, 20 ng/mL recombinant murine granulocyte-macrophage colony stimulating factor (GM-CSF), and 10 ng/mL recombinant murine Interleukin-4 (IL-4). Culture medium was replaced every 48 h. On day 7, loosely adherent BMDCs were harvested by gentle pipetting and centrifugation (450 × g, 5 min).

### 2.5 BMDCs maturation assessment

4T1 cells pretreated with phosphate-buffered saline (PBS) or DTP (12 h) received 650 nm laser irradiation (20 mW/cm^2^, 5 min). Then, BMDCs were co-cultured with DTP-PDT-treated 4T1 cells in Transwell^®^ inserts (0.4 μm pore, Corning) to enable soluble mediator exchange while preventing direct contact. Lipopolysaccharide (LPS; 1 mg/L) was used as a positive control. Following a 24-h co-culture period, the BMDCs were collected, labeled with anti-CD11c-BV421 (BioLegend, 117343), anti-CD80-PE (BioLegend, 104707), and anti-CD86-APC (BioLegend, 105011), and then subjected to flow cytometric analysis.

### 2.6 Cytokine detection

Cell supernatants from the transwell experiment were collected for cytokine detection using enzyme-linked immunosorbent assay (ELISA) kits specific for mouse tumor necrosis factor-α (TNF-α) (Cloud-Clone, SEA133Mu) and mouse interferon-γ (IFN-γ) (Cloud-Clone, SEA049Mu), strictly adhering to the protocols provided by the manufacturer.

### 2.7 Animals

Female BALB/c mice, aged 6–8 weeks (body weight 18–20 g), were purchased from HFK Bioscience (Beijing, China). Animals were maintained in a specific pathogen-free facility, regulated at constant temperature (22 ± 1°C), humidity (50% ± 10%), and a 12-h light/dark cycle. All animal experiments strictly followed the National Institutes of Health Guidelines for the Care and Use of Laboratory Animals. The Institutional Animal Care and Use Committee of the Institute of Radiation Medicine, Chinese Academy of Medical Sciences approved all experimental procedures under Ethics Approval No. IRM/2-IACUC-2409-094.

### 2.8 Evaluation of antitumor efficacy in a bilateral 4T1 tumor model

For bilateral breast tumor establishment, 5 × 10^5^ luciferase-transfected 4T1 (4T1-Luc) cells, suspended in 100 μL PBS, were first subcutaneously injected into the right flank to generate the primary tumor site. Once the primary tumor reached 60 mm^3^, a secondary challenge was developed by inoculating 1 × 10^5^ cells into the contralateral (left) flank to form a distinct distant tumor. Tumor growth was determined using the formula: Volume = (width^2^ × length) × 0.5.

When the primary tumor volumes reached 80–100 mm^3^, mice were randomly allocated into four treatment groups (n = 16 per group): Model (100 μL saline, intravenous), α-PD-L1 (10 mg/kg α-PD-L1 (A2115, Selleck, United States), administered intraperitoneal every 3 days), PDT (intravenous 10 mg/kg DTP followed by 650 nm laser irradiation at 100 J/cm^2^), and PDT + α-PD-L1 (combined DTP-PDT and α-PD-L1). Tumor dimensions and individual body weights were monitored every other day. Tumor progression was assessed weekly using bioluminescence imaging (IVIS Lumina III, Caliper Life Sciences).

### 2.9 Immune profiling and cytokine analysis

To evaluate the immune response, tumors, lnguinal lymph nodes, serum, and spleens were collected using sterile procedures on day 8 post-treatment. Tumor and lymphoid tissues underwent mechanical dissociation to generate single-cell suspensions. Subsequent processing included red blood cell (RBC) lysis, filtration, and extensive washing steps. Cell viability was determined using the Zombie Aqua™ Fixable Viability Kit (Biolegend, 423101). To block nonspecific binding, cells were incubated with TruStain FcX™ (anti-mouse CD16/32) antibody (Biolegend, 101319). Cells were then stained with the following antibodies: anti-CD45 FITC (Biolegend, 103107), anti-CD11c BV421 (Biolegend, 117329), anti-CD80 PE, anti-CD86 APC, anti-CD3ε Percp-cy5.5 (Biolegend, 100327), anti-CD4 PE (Biolegend, 100511), anti-CD8a APC (Biolegend, 100711), and anti-Forkhead box protein P3 (FoxP3) BV421 (Biolegend, 126419), followed by flow cytometric analysis. Serum levels of TNF-α and IFN-γ were measured using specific mouse ELISA kits. Immunofluorescence staining was performed on the tumor sections using anti-CD8 and anti-FoxP3 antibodies.

### 2.10 Histopathological assessment via hematoxylin and eosin (H&E) staining

On day 21 post-treatment, tumors were resected and fixed in 4% PFA, paraffin-embedded, and sectioned at 4 µm thickness. Following deparaffinization in xylene and graded ethanol rehydration, sections were subjected to H&E staining to evaluate the pathological changes. Images were acquired on a Leica DMILLED microscope system (Leica Microsystems). Ten fields per section were randomly selected for microscopic observation and quantitative analysis.

### 2.11 Statistical analysis

Data are expressed as mean ± standard deviation (SD). Data normality was confirmed via Shapiro-Wilk tests. Homogeneity of variance was validated using Levene’s test. For non-normally distributed data, Kruskal-Wallis with Dunn’s *post hoc* was employed. Differences between two groups were assessed via Student’s t-test, while differences among three or more groups were assessed via one-way analysis of variance (ANOVA). Statistical significance was defined as *p < 0.05, **p < 0.01, and ***p < 0.001.

## 3 Results

### 3.1 Optical properties of DTP

**FIGURE 2:**
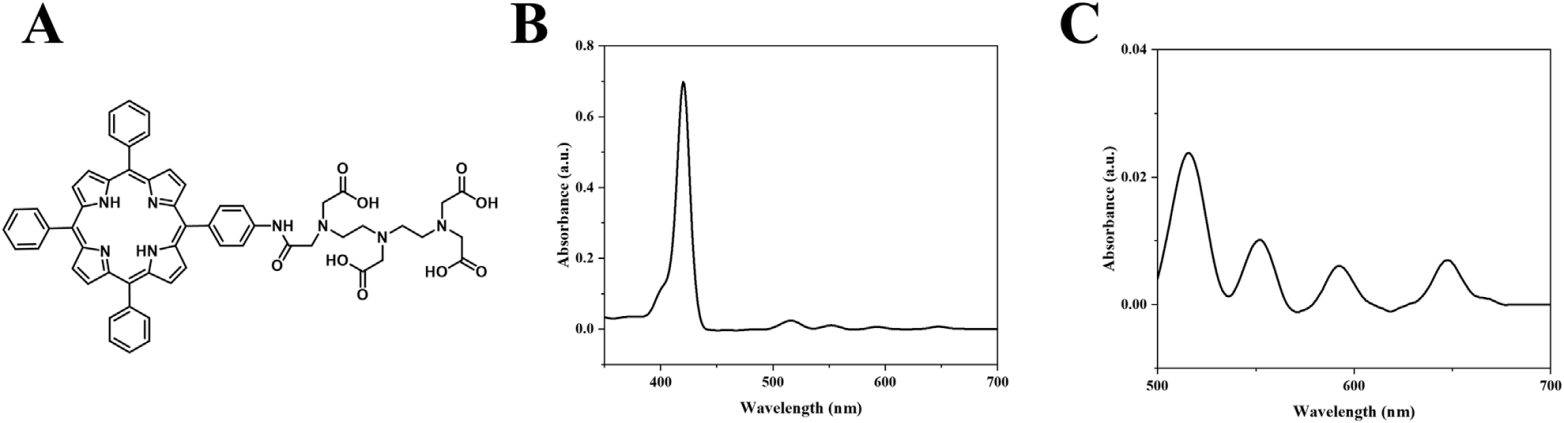
Structural and spectroscopic Analysis. **(A)** DTP molecular structure. **(B)** UV–Vis absorption spectrum of DTP from 350 to 700 nm (1.65 μM in DMSO). **(C)** Enlarged view of the 500–700 nm region from **(B)**.

The molecular structure of DTP is illustrated in Figure 2A. UV-Vis spectroscopy revealed the spectral profile of DTP, with the main peak at 420 nm and additional peaks at 516, 551, 592, and 647 nm (Figures 2B,C). While DTP exhibits a primary absorption peak at 420 nm, we specifically employed 650 nm laser irradiation during treatment to leverage its deeper tissue penetration capability in the red-light spectrum (Austin et al., 2021), following clinical PDT standards (Allison et al., 2024).

### 3.2 DTP-PDT induces ICD *in vitro*

**FIGURE 3:**
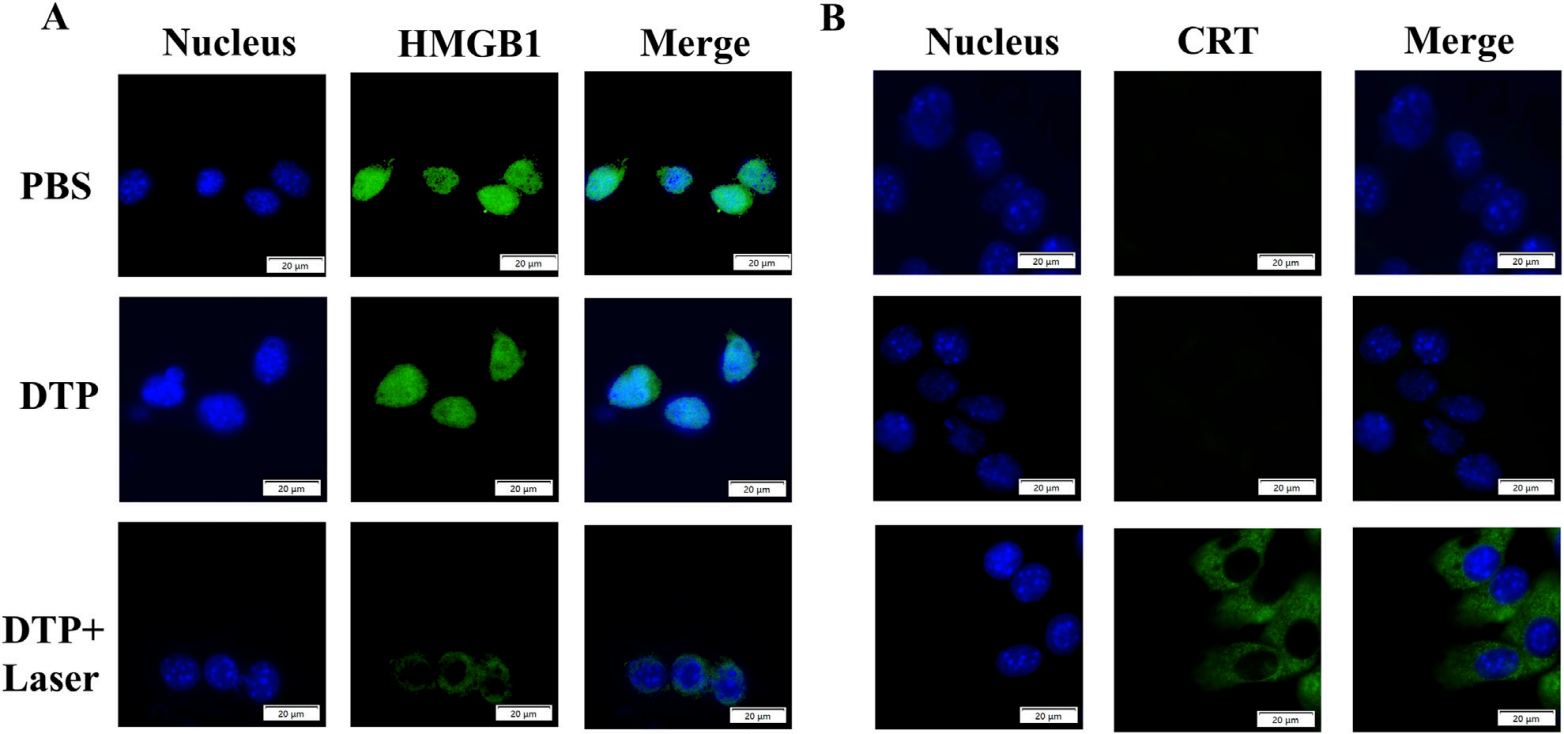
DTP-PDT induces ICD *in vitro*. Representative immunofluorescence images of HMGB1 release **(A)** and CRT membrane exposure **(B)** (green) in 4T1 cells following various treatments. DAPI (blue) labels nuclei. Scale bar = 20 μm.

After laser irradiation, 4T1 cells treated with DTP were subjected to immunofluorescence staining targeting HMGB1 and CRT to assess whether DTP-PDT could induce ICD. As shown in Figure 3A, the distribution of HMGB1 was found to be predominantly nuclear in PBS or DTP treated cells. In contrast, HMGB1 was released into the extracellular space in DTP-PDT-treated cells. As demonstrated in Figure 3B, the laser-treated group exhibited intense green fluorescence on the cell surface in comparison to the non-irradiated group, demonstrating considerably elevated levels of CRT exposure. These results suggest that DTP-PDT triggers HMGB1 release and CRT translocation, confirming the induction of ICD.

### 3.3 DTP-PDT triggers BMDCs maturation and cytokine release

**FIGURE 4:**
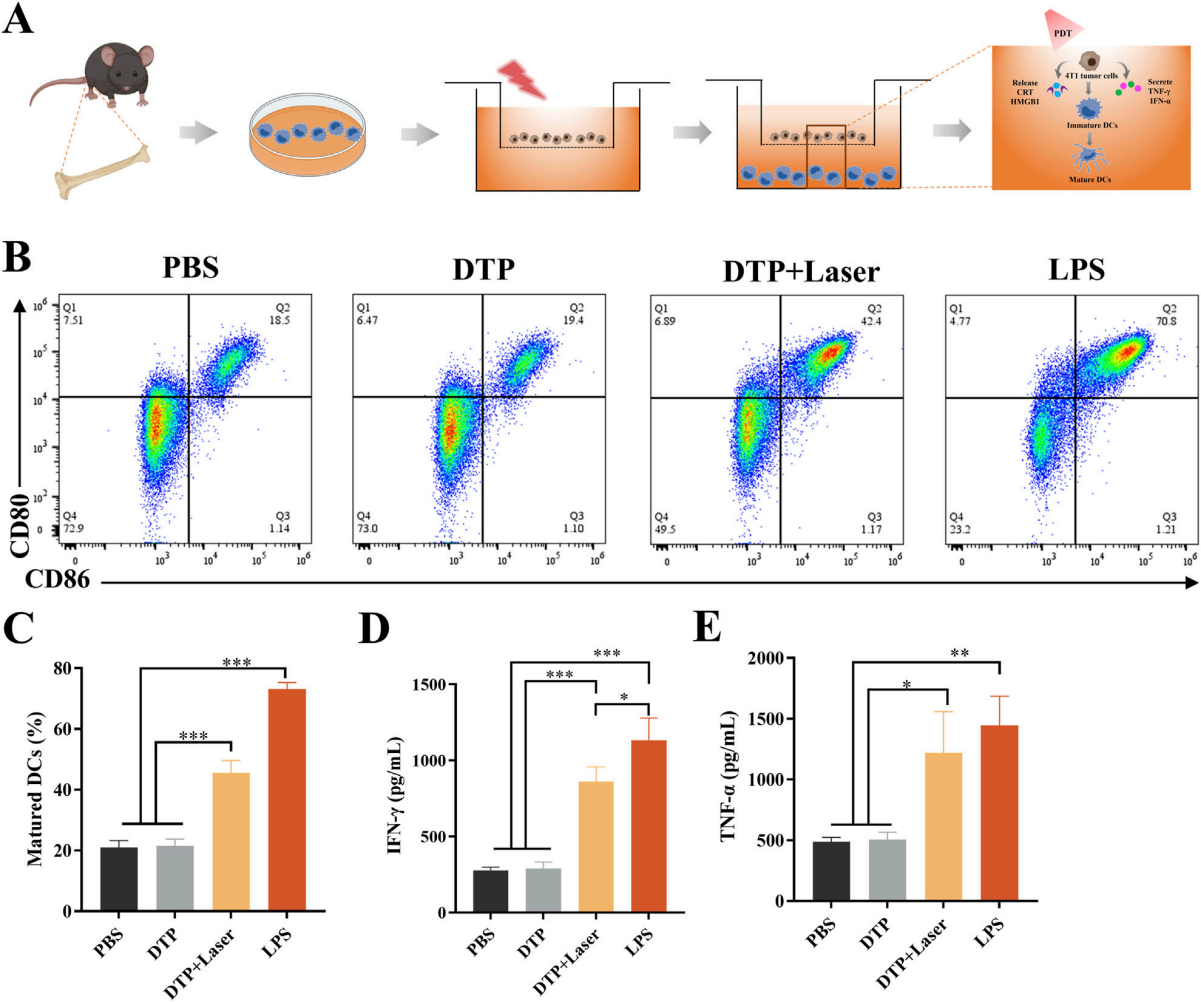
*In vitro* DC maturation and cytokine secretion induced by DTP-PDT. **(A)** Schematic design and mechanism of the transwell co-culture system: Treated 4T1 cells (upper chamber) and BMDCs (lower chamber). **(B)** Proportion of mature BMDCs (CD11c^+^CD80^+^CD86^+^) following co-culture with DTP-PDT-treated 4T1 cells. **(C)** Quantification of the proportion of mature BMDCs from transwell system experiments (n = 3). **(D,E)** ELISA quantification of secreted **(D)** IFN-γ and **(E)** TNF-α in culture supernatants (n = 3). Data are shown as mean ± SD. *p < 0.05, **p < 0.01, ***p < 0.001.

BMDCs were co-cultured with DTP-PDT-treated 4T1 cells using the experimental design illustrated in Figure 4A. Following co-culture, BMDCs exhibited significant maturation, as evidenced by upregulated expression of co-stimulatory markers CD80 and CD86. As demonstrated in Figures 4B,C, DTP + Laser induced a significantly higher proportion of mature BMDCs (45.53% ± 4.12%) in comparison with PBS (21% ± 2.29%) and DTP (21.57% ± 2.20%) groups (*p* < 0.001).

Specifically, significant increases in the secretion of IFN-γ (*p* < 0.001, Figure 4D) and TNF-α (*p* < 0.05, Figure 4E) were observed in the DTP + Laser group compared to the PBS and DTP groups. These findings indicate that DTP-PDT effectively induces DAMP release, promotes DC maturation, and enhances the expression of pro-inflammatory cytokines, thereby initiating immune responses.

### 3.4 Anti-abscopal effect of DTP-PDT combined with α-PD-L1 therapy in bilateral 4T1 tumor model

**FIGURE 5:**
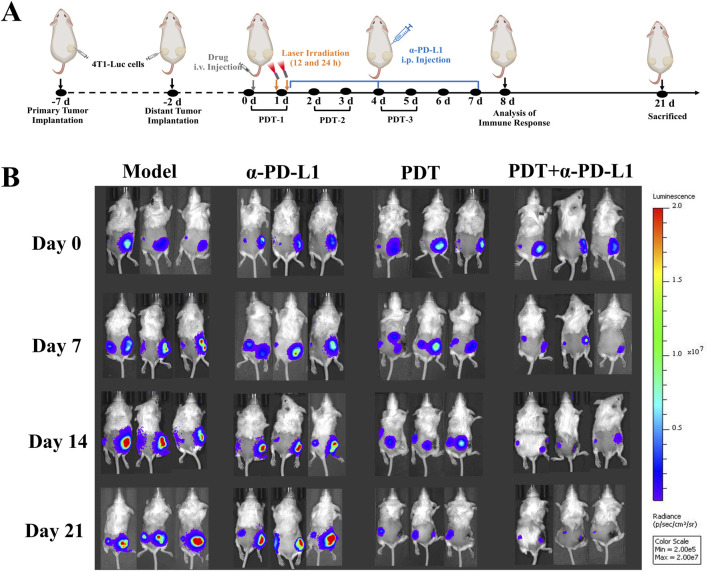
Bilateral Tumor Model Design and Therapeutic Monitoring **(A)** The experimental design in the bilateral 4T1 tumor model. Primary tumors were treated locally with DTP-PDT (650 nm laser, 100 J/cm^2^), while contralateral tumors remained untreated. α-PD-L1 (10 mg/kg) was administered intraperitoneally (i.p.) every 3 days. **(B)** *In vivo* bioluminescence imaging of tumor-bearing mice on days 0, 7, 14, and 21.

**FIGURE 6:**
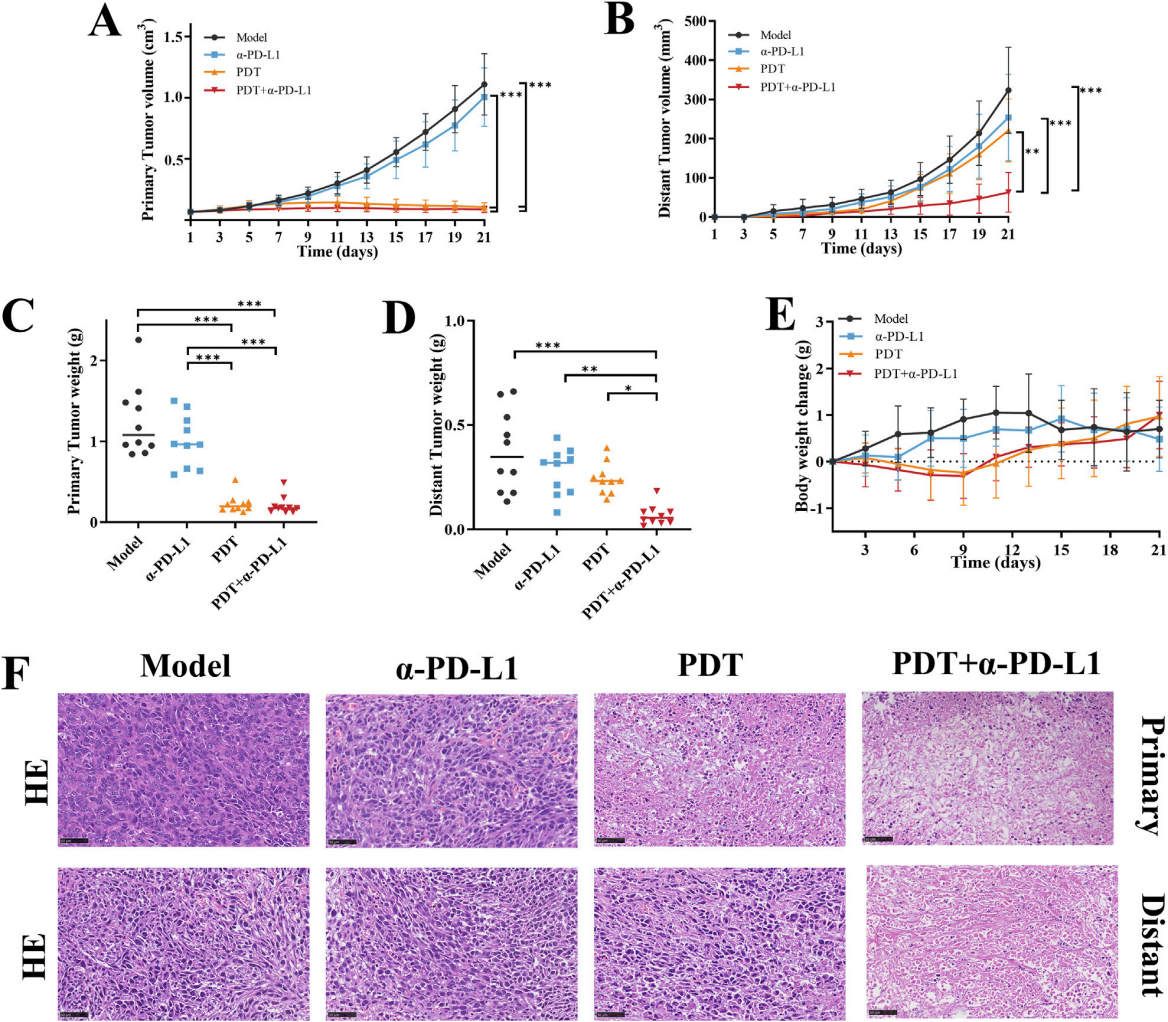
The antitumor efficacy of DTP-PDT in combination withα-PD-L1 therapy. Growth curves of **(A)** primary and **(B)** distant tumors following different treatments (n = 10 mice/group). **(C,D)** Terminal tumor weights at Day 21 (n = 10 mice/group). **(E)** Changes in mouse body weight over the 21-day therapy (n = 10 mice/group). **(F)** Representative images of H&E-stained primary and distant tumor sections. Scale bar = 50 μm. Data are shown as mean ± SD. *p < 0.05, **p < 0.01, ***p < 0.001.

To address the limited efficacy of α-PD-L1 monotherapy in TNBC, we assessed the potential of DTP-PDT-induced ICD to enhance α-PD-L1 therapy and improve systemic antitumor efficacy in the 4T1 breast cancer model (Figure 5A). In the α-PD-L1 monotherapy group, local and distant tumor growth showed no significant difference compared with the model group (Figures 5B, 6A,B). In contrast, DTP-PDT alone resulted in an 81.62% reduction in primary tumor weight (*p* < 0.001 vs. model and α-PD-L1), but only a 35.35% reduction in distant tumor weight (*p* > 0.05 vs. model and α-PD-L1), highlighting that PDT has a significant therapeutic effect on irradiated primary tumors but little effect on unirradiated distant tumors. Remarkably, combining DTP-PDT and α-PD-L1 demonstrated a significant abscopal effect, with 83.31% primary tumor regression (*p* < 0.001 vs. model and α-PD-L1) and 82.15% distant tumor suppression (*p* < 0.001 vs. model, *p* < 0.01 vs. α-PD-L1, *p* < 0.05 vs. PDT) (Figures 6C,D). Importantly, across all treatment groups, the body weight change revealed no significant difference which suggests that the combined strategy has a good tolerability (Figure 6E).

Following treatment, H&E staining was performed on the primary and distant tumor tissue to observe histopathological changes. Figure 6F illustrates that tumor cells in the model and α-PD-L1 groups, as well as distant tumors in the PDT group, exhibited tight cell arrangements with rounded nuclei and well-defined nucleoli. In contrast, tumors in the PDT + α-PD-L1 group and primary tumors in the PDT group, displayed extensive nuclear consolidation, nuclear fragmentation, and visible necrotic areas. These findings provide further evidence of the remarkable inhibitory effect of the combination therapy on primary and distant tumors.

Collectively, our findings suggest that DTP-PDT-induced tumor-specific immune responses may be effective in sensitizing tumors towards PD-L1 blockade, and combining DTP-PDT with PD-L1 blockade could be a promising approach in treating metastatic TNBC.

### 3.5 Combined therapy reprograms the local tumor immune microenvironment

**FIGURE 7:**
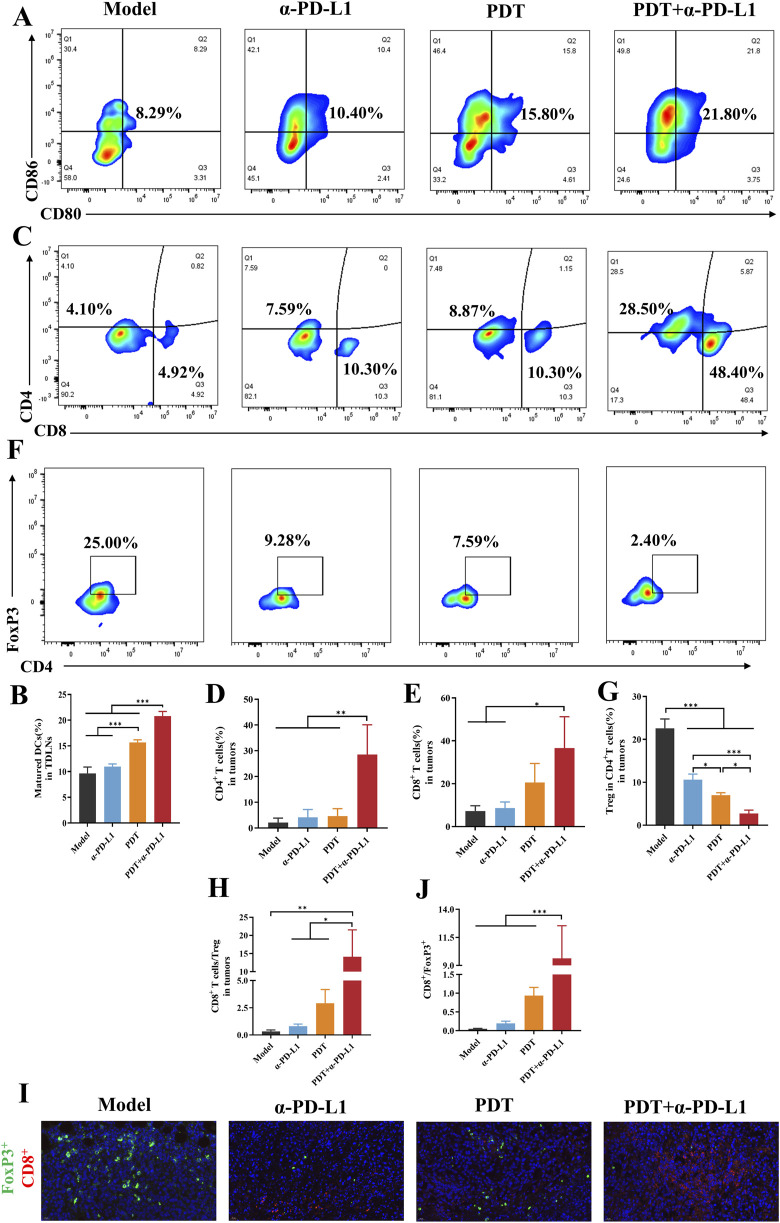
DTP-PDT in combination with α-PD-L1 therapy for TME reprogramming. **(A,B)** The proportion of mature DCs in lymph nodes (gated on CD11c^+^ cells). **(C–E)** The proportion of T cells in distant tumors (gated on CD3^+^ T cells). **(F,G)** The proportion of Tregs in distant tumors (gated on CD3^+^CD4^+^ cells). **(H)** The CD8^+^ T cells ratio to Tregs in distant tumors. **(I)** Representative immunofluorescence images of distal tumors stained for CD8^+^ (green), FoxP3^+^ (red), and DAPI (blue). Scale bar = 20 μm. **(J)** Quantitative analysis of **(I)**. Data are shown as mean ± SD. *p < 0.05, **p < 0.01, ***p < 0.001.

The synergistic antitumor effects observed in the combination therapy prompted further investigation of its immunological mechanisms. In order to assess the immune response, we analyzed populations of immune cells in the lymph nodes, tumors, and spleens. In our study, DC maturation was evaluated by flow cytometry in tumor-draining lymph nodes. As shown in Figures 7A,B, the DTP-PDT and DTP-PDT+α-PD-L1 groups had a significantly higher percentage (15.8% and 21.8%, respectively) of mature DCs (CD45^+^CD11c^+^CD80^+^CD86^+^) compared to the model (8.29%) and α-PD-L1 (10.40%) groups (*p* < 0.001), indicating that PDT promotes DC maturation *in vivo*.

The analysis of tumor-infiltrating T cells was performed in distant tumors. DTP-PDT+α-PD-L1 group revealed a 9.8-fold and 6.9-fold increase in CD8^+^ and CD4^+^ T cells, respectively, compared to the model group, indicating efficient CTLs infiltration (Figures 7C–E). In contrast, neither the DTP-PDT nor the α-PD-L1 groups showed significant differences in T cell subpopulations (*p* > 0.05) relative to the model group, indicating that monotherapy was insufficient to elicit an immune response. Moreover, the proportion of Tregs (CD3^+^CD4^+^FoxP3^+^) in the combined treatment group significantly decreased compared to that in the model (*p* < 0.001) and PDT (*p* < 0.05) groups, confirming a reduction in tumor-associated immunosuppression (Figures 7F,G). And then the ratio of CD8^+^ T cells to Tregs were compared and a significant increase could be observed in Figure 7H (*p* < 0.01 vs. model, *p* < 0.05 vs. α-PD-L1 and PDT). Subsequently, immunofluorescence staining was performed on distant tumor sections to characterize the tumor immune microenvironment, Figure 7I revealed that more red fluorescence and less green fluorescence were observed in the combined therapy group, indicating the increased CD8^+^ T cell infiltration and decreased FoxP3^+^ expression. Quantitative analysis demonstrated a significant increase of CD8^+^/FoxP3^+^ ratio in the PDT + α-PD-L1 group (9.63 ± 2.91) relative to other groups (*p* < 0.001, Figure 7J).

### 3.6 Combined therapy induces systemic immune activation

**FIGURE 8:**
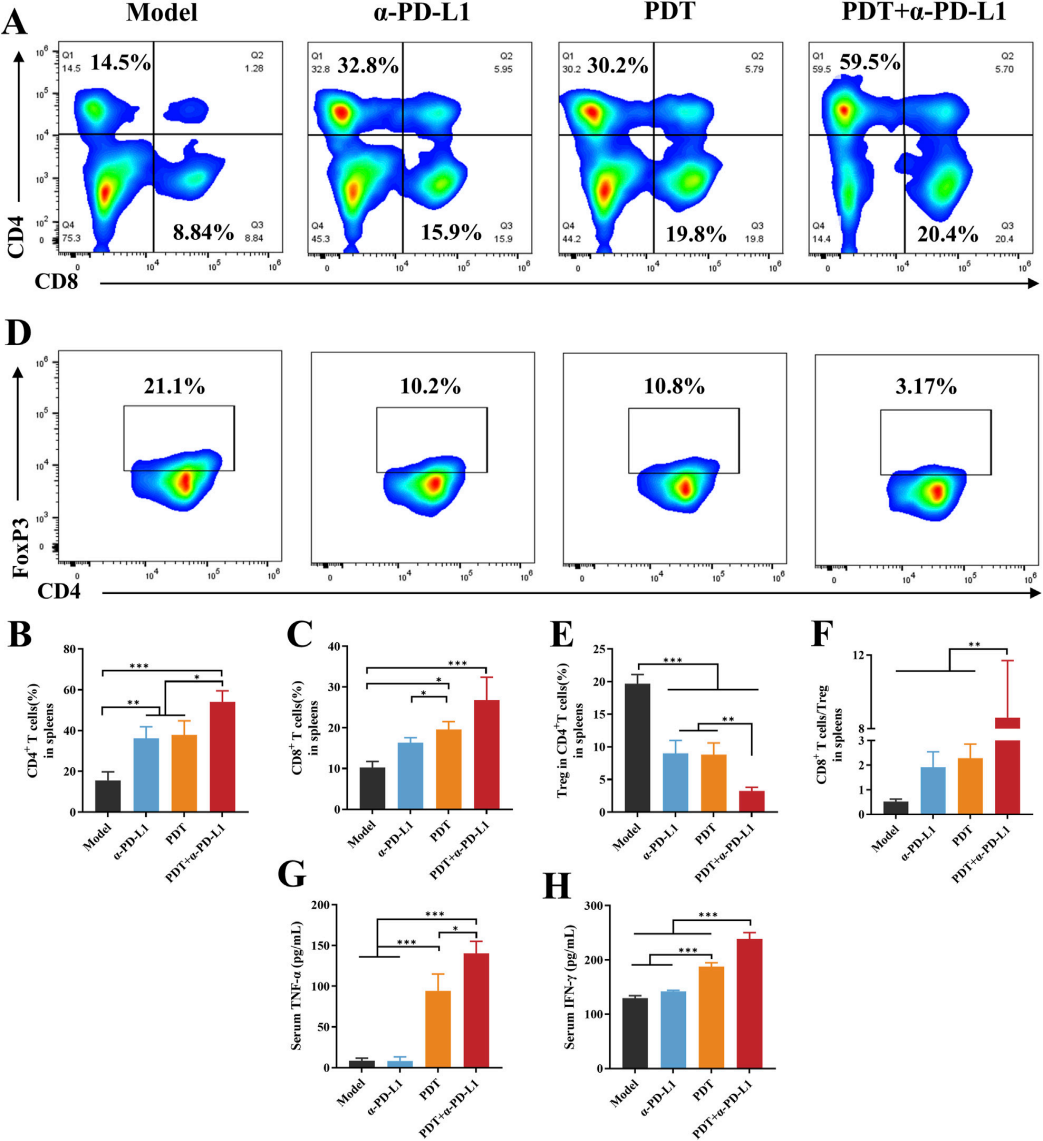
DTP-PDT in combination withα-PD-L1 therapy for systemic immune activation. **(A–C)** The proportion of T cells in spleens (gated on CD3^+^ T cells). **(D,E)** The proportion of Tregs in spleens (gated on CD3^+^CD4^+^ cells). **(F)** The CD8^+^ T cells ratio to Tregs in spleens. **(G–H)** Levels of cytokines **(G)** TNF-α and **(H)** IFN-γ in serum. Data are shown as mean ± SD. *p < 0.05, **p < 0.01, ***p < 0.001.

Systemic immune activation was evaluated by splenic T cell redistribution. Figures 8A–C revealed DTP-PDT monotherapy elevated CD8^+^ and CD4^+^ T-cell proportions by 2.2-fold and 2.1-fold *versus* Model (*p* < 0.05). Notably, the combination therapy group showed a statistically higher CD4^+^ T cell proportion relative to either PDT or α-PD-L1 monotherapy. CD8^+^T cells proportion also increased considerably in the DTP-PDT group compared to both model and α-PD-L1 groups (*p* < 0.05), indicating that PDT enhances the differentiation of naive T cells into CD8^+^ T cells. The proportion of splenic Treg was significantly suppressed in the combined group (*p* < 0.001 vs. model, *p* < 0.01 vs. α-PD-L1 and PDT) (Figures 8D,E). Furthermore, the ratio of CD8^+^ T cells to Tregs was significant increased compared to other groups (Figure 7F, *p* < 0.05).

PDT-mediated tumor cell death induces local inflammation, accompanied by cytokine secretion, particularly TNF-α and IFN-γ (Evans et al., 1990; Zhang et al., 2022). TNF-α increased remarkably in the groups of PDT and combined therapy in comparison with the model and α-PD-L1 groups (*p* < 0.001, Figure 8G). TNF-α levels were significantly elevated in the combination treatment group compared to the PDT group (*p* < 0.05). IFN-γ was also upregulated in the combination group when compared to the other groups (*p* < 0.001, Figure 8H).

Collectively, these results suggested that combining DTP-PDT and α-PD-L1 therapy reshapes the immunosuppressive TME by enhancing T cell infiltration, promoting antitumor cytokine production and limiting immunosuppressive cells. This provides a strong biological foundation for the observed abscopal effects in the mTNBC model.

## 4 Discussion

Our study represents the first demonstration that DTP-PDT induces typical ICD features, including the surface exposure of CRT, release of HMGB1, and secretion of pro-inflammatory cytokines, all of which significantly promotes DC maturation. In a bilateral TNBC model, the synergistic treatment of DTP-PDT and α-PD-L1 substantially suppressed untreated distant tumors through systemic immune potentiation. This therapeutic effect was linked to elevated CD8^+^ and CD4^+^ T lymphocyte infiltration, and decreased proportion of Treg in both the TME and spleen.

The release of DAMPs is a critical event in ICD (Fucikova et al., 2020). Surface-translocated CRT functions as an “eat-me” signal, promoting DCs to phagocytose tumor antigens (Fucikova et al., 2020). Meanwhile, HMGB1 release activates DCs via the Toll-like receptor 4 signal pathway (Krysko et al., 2012). These events promote DC maturation (Alzeibak et al., 2021), and stimulate cytokine secretion, particularly IFN-γ and TNF-α (Andersson et al., 2000; Zhou et al., 2019), which further enhance DC function. IFN-γ enhances DC antigen presentation efficiency through major histocompatibility complex class I (MHC-I) molecules (Frucht et al., 2001; Todorović-Raković, 2022), while TNF-α further promotes DC maturation and migration capacity (Salazar-Onfray et al., 2007). Together, these cytokines may sustain and amplify DC activation, synergistically boosting the antitumor immune response initiated by DTP-PDT.

We used a bilateral 4T1 tumor model to simulate mTNBC progression. In this model, the primary tumor treated with DTP-PDT and the contralateral untreated tumor represented metastasis. Results showed minimal inhibitory effect against both primary and distant tumors in α-PD-L1 monotherapy group, validating the intrinsic immune-resistance of the 4T1 model. This resistance is primarily attributed to the low tumor mutational burden and high immunosuppressive TME of 4T1 tumor (Kim et al., 2014; Abe et al., 2016). Our analyses revealed impaired antigen presentation in 4T1 tumor model, evidenced by reduced mature DC proportions in TDLNs. Furthermore, the levels of CD4^+^ and CD8^+^ T cell infiltration in distant 4T1 tumors are low, while the proportion of Tregs is high, suggesting a dominant immunosuppressive TME. Crucially, the combination of DTP-PDT and α-PD-L1 therapy achieved significant distant tumor control. This indicates that local DTP-PDT-induced ICD can stimulate systemic antitumor immunity, eliciting an abscopal effect that inhibits metastatic lesions. This provides a potential therapeutic strategy for treating metastatic TNBC.

Immunologically, the combination therapy enhanced the maturation of DCs and overcame the inherent antigen presentation defects in TNBC. With the increase of CD8^+^ T cell infiltration and the decrease in the proportion of Tregs in distant tumors, the TME has shifted from an immunosuppressive “cold” state to a more immunogenic “hot” state. Notably, one of the important effector cells in the “hot” microenvironment of this transition is tumor-infiltrating lymphocytes (TILs), which contain a variety of immune system cells. Among them, the expansion of CD8^+^ T cells directly mediates tumor cell killing (St. Paul and Ohashi, 2020), while CD4^+^ T cells likely support this response (Kervevan and Chakrabarti, 2021). Tregs are the most representative immunosuppressive cells among TILs, which express the transcription factor FoxP3 and negatively regulate anticancer immunity (Kanamori et al., 2016). The reduction in Treg percentage may be partly due to the local inflammation induced by DTP-PDT, which potentially suppresses Treg differentiation. Additionally, CD8^+^ T cell-derived IFN-γ may further inhibits Treg function, potentially via FoxP3 downregulation. Furthermore, the ratio of CD8/Treg is a more sensitive indicator of immune function rather than evaluation of Treg or CD8^+^ T alone. In this study, this ratio was significantly increased in the combination therapy group and the PDT group, suggesting that these treatments induced an effective antitumor immune response and may also be associated with improved prognosis (Liu et al., 2011; Goda et al., 2022). At the systemic immunity level, the expansion of both CD8^+^ and CD4^+^ T cells, as well as a reduction in Tregs in the spleen, suggest the establishment of systemic antitumor immunity that is essential for controlling early micro-metastases in TNBC.

Collectively, the synergy between DTP-PDT and PD-L1 blockade achieves its therapeutic effect on metastatic TNBC through the following mechanism. Local tumor cell death caused by DTP-PDT results in the release of tumor associated antigens and DAMPs. These DAMPs activate infiltrating dendritic cells (DCs), promoting their phagocytosis of tumor antigens and driving their maturation and subsequent migration to tumor-draining lymph nodes. There, mature DCs present the antigens to activate naïve T cells, thereby initiating antigen-specific CD8^+^ cytotoxic and CD4^+^ helper T cell responses. PD-L1 blockade alleviates T cell exhaustion, enhancing T cell infiltration into distant tumors. The activated CD8^+^ T cells kill tumor cells directly, while CD4^+^ T cells support this process, ultimately leading to the suppression of distant tumors.

However, the present study does exist some limitations. (1) Our current findings demonstrate abscopal effect following combination therapy, we recognize the critical need to investigate the durability of these therapeutic effects. Future studies should systematically assess long-term survival outcomes, particularly the advanced recurrences after treatment and conduct tumor rechallenge experiments to the establishment of protective immune memory. (2) The 4T1 model used in this study includes key features of human TNBC metastasis, but its murine origin and tumor microenvironment differ from human pathophysiology. Future validation in patient-derived xenografts or humanized models will be essential for clinical translation.

## Data Availability

The raw data supporting the conclusions of this article will be made available by the authors, without undue reservation.
